# Broad-Spectrum and Gram-Negative-Targeting Antibiotics Differentially Regulate Antibody Isotype Responses to Injected Vaccines

**DOI:** 10.3390/vaccines9111240

**Published:** 2021-10-25

**Authors:** Aklilu F. Haile, Rachel M. Woodfint, Eunsoo Kim, Marisa R. Joldrichsen, Nega Berhe, Wondwoossen A. Gebreyes, Prosper N. Boyaka

**Affiliations:** 1 Department of Veterinary Biosciences, The Ohio State University, Columbus, OH 43210, USA; aklilu.feleke@aau.edu.et (A.F.H.); woodfint.1@buckeyemail.osu.edu (R.M.W.); Kim.3772@osu.edu (E.K.); joldrichsen.1@buckeyemail.osu.edu (M.R.J.); 2 Aklilu Lemma Institute of Pathobiology, Addis Ababa University, Addis Ababa 1000, Ethiopia; nega.berhe@aau.edu.et; 3 Department of Preventive Medicine, The Ohio State University, Columbus, OH 43210, USA; gebreyes.1@osu.edu; 4 Global One Health Initiative, The Ohio State University, Columbus, OH 43210, USA; 5 Infection Diseases Institute, The Ohio State University, Columbus, OH 43210, USA; 6 Department Microbial Immunity and Infection, The Ohio State University, Columbus, OH 43210, USA

**Keywords:** antibiotics, IgA responses, vaccines

## Abstract

Antibiotics are extensively used worldwide for the treatment of common infections by agents such as *E. coli* and *Salmonella.* They also represent the most common cause of alteration of the microbiota in people. We addressed whether broad-spectrum and Gram-negative-targeting antibiotics differentially regulate systemic and mucosal immune responses to vaccines. Antibiotics treatment enhances serum IgG1 responses in mice immunized systemically with a model polyvalent vaccine. This increase was not seen for other IgG subclasses and was dependent on the immunogenicity of vaccine antigens. The broad-spectrum antibiotic cocktail also enhanced serum IgA responses. Interestingly, both the broad spectrum and the antibiotic targeting Gram-negative bacteria enhanced the number of IgA antibody secreting cells in the intestinal lamina propria. This effect was unlikely to be due to an increase in cells expressing gut-homing receptors (i.e., CCR9 and α_4_β_7_) in peripheral tissues. On the other hand, the microbiome in mice treated with antibiotics was characterized by an overall reduction of the number of firmicutes. Furthermore, *Bacteroidetes* were increased by either treatment, and *Proteobacteria* were increased by the broad-spectrum antibiotics cocktail. Thus, immunoglobulin isotype and subclass responses are differentially regulated by oral antibiotics treatment and the gut microbiota shapes mucosal antibody responses after systemic immunization.

## 1. Introduction

Commensal microbes play an important role as regulators of health. It is now well-established that the beneficial effects of the microbiota require a balanced microbial community consisting of hundreds of different microbial species including bacteria, viruses, eukaryotic microbes, and archaea [1,2,3,4]. The most abundant of those organisms are bacteria of the *Bacteroidetes* (Gram-negative), *Actinobacteria* (Gram-positive)*,* and *Firmicutes* (Gram-positive) phyla [5]. Conversely, dysbiosis, or altered microbiota, has been associated with a variety of non-infectious diseases including allergy, asthma, diabetes, inflammatory bowel disease, obesity, cancer, and autism [6,7,8,9]. Compelling evidence also show that the microbiota plays a major role in host susceptibility to infectious diseases [10,11].

Vaccines have proven to be the more effective tools in the fight against infection by microbial pathogens. In fact, successful vaccination can induce antigen-specific antibodies that will block the diffusion of the bacterial or viral pathogens throughout the body or neutralize their virulence factors. Vaccination can also induce the needed CD4^+^ T cell responses to support these antibody responses and CD8^+^ T cell responses that provide protection against intracellular pathogens. Most current vaccines are injected vaccines which induce good antibody responses, mostly IgG, in the systemic compartment. However, induction of IgA responses in mucosal tissues, and thus provision of an additional layer of protection at the portal of entry of pathogens, typically requires immunization via mucosal route (i.e., oral, nasal, or sublingual) [12,13]. The recent development of RNA vaccines against SARS-CoV-2 is a testimony of the progress made in the development of vaccine adjuvants and delivery systems [14,15,16]. Despite these advances, the magnitude and profile of immune responses to immunization are substantially variable among individuals. For example, the immature immune system in infants or the aging immune system in the elderly are known factors that influence the efficacity of vaccines in these population. Diet and geographic location were more recently identified as determinants of the potential variability in the response to vaccination. This notion is supported by several studies showing that vaccines are often less effective at generating protective immunity in developing countries where malnutrition and infectious diseases are more prevalent [17,18,19].

The composition of the intestinal microbiota is increasingly emerging as a major driver of the efficacity of vaccines [20,21,22]. Furthermore, members of the nasal [23] and gut microbiota [24] were reported to influence the generation of vaccine-specific IgA responses after immunization with a live attenuated influenza vaccine or the polio vaccine, respectively. Antibiotics are extensively used worldwide for the treatment of infections. They also alter the microbiota [9] and thus, can reformat immune response to vaccines [25]. Antibiotics can broadly be segregated according to the type of bacteria (i.e., Gram-negative or Gram-positive) targeted. Infections by the Gram-negative bacteria *E. coli* and *Salmonella* remain important public health issues [26,27,28] and are major drivers of antibiotics use, especially in less-developed countries. We addressed whether vaccine efficacy will be impaired if it is administered during the course of treatment with antibiotics. More specifically, we compared the modulatory effect of an antibiotics targeting Gram-negative bacteria with that of a broad-spectrum antibiotics cocktail on systemic and mucosal immune responses in a mouse model of multivalent vaccine.

## 2. Materials and Methods

### 2.1. Mice

C57BL/6 mice were obtained from The Jackson Laboratory (Bar Harbor, ME, USA) and maintained under specific pathogen-free conditions at The Ohio State University animal care facility. All animal experiments were approved by the Institutional Animal Care and Use Committee and followed the federal guidelines to avoid unnecessary pain and suffering. All experiments were in accordance with both National Institutes of Health and Institutional Animal Care and Use Committee guidelines to avoid pain and distress.

### 2.2. Antibiotics Treatment

**Figure 1:**
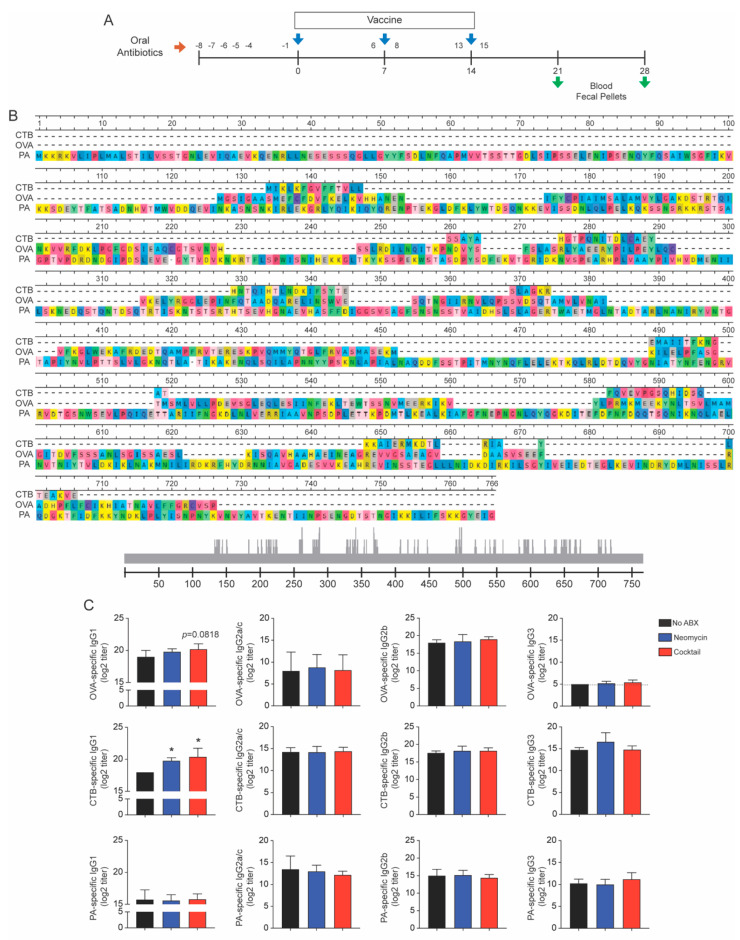
The immunogenicity of unrelated vaccine antigens is differentially affected by oral treatment with broad spectrum- and Gram-negative-targeting antibiotics. (**A**) Experimental scheme. (**B**) Multiple alignment comparing amino acid sequences of the vaccine antigens ovalbumin (OVA), protective antigen of anthrax (PA), and B subunit of cholera toxin (CTB) which also served as adjuvant. Gray scale at the base indicates amino acid position and conserved areas within the sequences. (**C**) Antigen-specific IgG subclass responses. Mice were orally treated with antibiotics and then immunized weekly intervals on days 0, 7, and 14 by intraperitoneal injection of the multivalent vaccines. Additional doses of antibiotics were orally administered 24 hours before and 24 hours after vaccination. Serum samples were collected a week after the last immunization (day 21) and antigen-specific IgG1, IgG2a/c, IgG2b, and IgG3 subclass titers measured by ELISA. Data are expressed as mean Ab titers ± SD (*n* = 5 mice per group) and are from three independent experiments. * *p*  ≤ 0.05 compared to controls non-treated with antibiotic (No ABX).

Mice were divided in three groups based on antibiotics treatment (none, Gram-negative and broad antibiotics treatment groups). One antibiotic regimen consisted of neomycin (1 mg/mL) which targets Gram-negative bacteria. The broad antibiotic treatment consisted of a cocktail of different antibiotics including ampicillin (1 mg/mL), vancomycin (0.5 mg/mL), neomycin (1 mg/mL), gentamicin (1 mg/mL), and metronidazole (1 mg/mL). Antibiotics were administered orally using gavage (200 μL/mouse) on days -8, -7, -6, -5, and -4 before the first immunization (day 0). Additional doses were given one day before and one day after each immunization (see Figure 1A). Freshly emitted fecal pellets were collected before (controls) and after antibiotic treatments (at day 16) for the analysis of gut microbial community (see Figure 1A).

### 2.3. Immunization and Sample Collection

Mice were immunized 3 times, one week apart (days 0, 7, and 14), by intraperitoneal injection of a 100 μL of saline containing combination of vaccine antigens (100 μL/mouse). The antigens consisted of 20 μg of ovalbumin (OVA, grade V, Sigma, St. Louis, MO, USA), 20 μg of recombinant cholera toxin B subunit (CTB) and 20 μg of *Bacillus anthracis* protective antigen (PA). Blood samples were collected one week after the last immunization (day 21) to monitor the magnitude of Ag-specific Ab responses. Fecal extracts were collected on days 21 and 28 as previously described for assessment of mucosal S-IgA Ab responses [29].

### 2.4. Titration of Antigen-Specific IgG and IgA Abs

To determine Ag-specific antibody titers, ELISA was performed with Ag-coated plates as described previously [29,30,31,32]. Briefly, microtiter plates were coated with Ag (5 μg/mL for CTB and PA, or 1 mg/mL for OVA). For detection of Ag-specific IgG and IgA Abs, serial dilutions of serum or fecal material extract were added to the plates and the binding antibodies were detected with HRP-conjugated anti-mouse µ- or γ-heavy chain-specific antisera (Southern Biotech Associates Inc., Birmingham, AL). Biotin-conjugated rat anti-mouse IgG1, IgG2a/c, IgG2b or IgG3 monoclonal Abs (mAbs) and HRP-conjugated streptavidin (BD Bioscience, San Jose, CA, USA) were used to measure IgG subclass responses. The Ab titers were determined as the last dilutions of samples with an absorbance of >0.1 above that of control samples from naive mice. For assessment of IgA levels in the intestinal secretions, freshly emitted fecal pellets were normalized by weight and homogenized in PBS (1 mL per 0.1 g feces). After centrifugation, dilutions of supernatants were used for evaluation of antigen-specific IgA levels as described above.

### 2.5. Antibody Secreting Cell (ASC) Responses

For assessment of cells secreting immunoglobulin isotypes, Peyer’s patches and lamina propria were collected from antibiotic-treated and control mice. The frequencies of antibody secreting cells (ASC) in single cell suspensions were analyzed by immunoglobulin isotopes ELISPOT assay [33].

### 2.6. Flow Cytomety Analysis

Expression of gut-homing receptors by B cells, T cells and myeloid cells was analyzed by flow cytometry. Briefly, suspensions of spleen and mesenteric lymph node cells were stained with antibodies anti-α_4_β_7_, -CCR9, -CD4, -CD8, -CD11b, and CD19. Stained cells were analyzed with an Attune NxT flow cytometer (Thermo Fisher Scientific, Waltham, MA, USA).

### 2.7. Analysis of Gut Microbiota

Samples were collected twice a day from each individual mouse and pooled to minimize potential daily variation of the microbiota. Bacterial DNA was extracted by conventional methods (Qiagen, Valencia, CA, USA), and 16S rRNA genes were amplified with the modified 16S eubacterial primers 28F, 5′-GAG TTT GAT CNT GGC TCA G-3′ and 519R, 5′-GTN TTA CNG CGG CKG CTG-3′ for amplifying the 500 bp region of 16S rRNA genes, as previously described [30,34]. The sequences were clustered into operational taxonomic units (OTU) with 96.5% identity (3.5% divergence) using USEARCH, and the seed sequence was put into a FASTA-formatted sequence file. The FASTA files were then queried against a database of high-quality sequences derived from NCBI using a distributed .NET algorithm that utilizes BLASTN+ (KrakenBLAST www.krakenblast.com, accessed on 21 March 2020). Principal component analysis (PCA) was used to summarize the relationship between microbial communities in the control and Cd-treatment groups. Linear discriminant analysis (LDA) scores were analyzed using the Galaxy software (https://huttenhower.sph.harvard.edu/galaxy/, accessed on 3 May 2020) and the threshold on the logarithmic for discriminative features was set at >2.5.

### 2.8. Multiple Alignment of Amino Acid Sequences

The open-source bioinformatics software, UGENE, was utilized for the analysis of the amino acid sequences of OVA, PA, and CTB. The exported sequences were formatted as CLUSTALW files and arranged using the public domain MUltiple Sequence Comparison by Log-Expectation (MUSCLE) software.

### 2.9. Statistical Analysis

Results were expressed as mean ± one standard deviation. Statistical significance was determined by one-way ANOVA, followed by Tukey post-hoc test. All statistical analyses were performed with the StataSe 12.0 software (StataCorp LLC, College Station, TX, USA) and GraphPad Prism 7 (GraphPad Software Inc, La Jolla, CA, USA). Results were considered significant at *p* < 0.05.

## 3. Results

### 3.1. Oral Antibiotics Treatment Differentially Regulates Ig Subclass Responses to A Multivalent Injected Vaccine

Several studies suggest that antibiotics regulate host immune responses to vaccines although this notion has been challenged by other studies, and reasons for these discrepancies are not well understood. We addressed whether an antibiotic targeting Gram-negative bacteria (i.e., neomycin) and broad-spectrum antibiotic cocktail differentially regulated immune responses to vaccine antigens. In order to model multivalent vaccines containing several unrelated vaccine antigens, mice were immunized by i.p. injection of ovalbumin (OVA), protective antigen (PA) of *Bacillus anthracis* and the B subunit of cholera toxin (CTB) (Figure 1A). As depicted in Figure 1B, OVA, PA, and CTB only share minimum amino acid sequences and thus, were unlikely to induce significant cross-reactive antibody responses.

Analysis of antigen-specific IgG subclass responses showed that oral treatment with either the Gram-negative targeting antibiotics or the broad-spectrum cocktail of antibiotics differentially affected IgG responses to different antigens. Thus, it significantly enhanced CTB-specific IgG1 responses (Figure 1C). The broad-spectrum cocktail of antibiotics also enhanced OVA-specific IgG1 responses, but neither treatment enhanced PA-specific IgG1 responses (Figure 1C). No other IgG subclass response was affected by the antibiotics treatment.

Together, these data show that the regulatory effect of antibiotics treatment on serum IgG responses to vaccine is dependent on the nature and/or immunogenicity of the vaccine antigens.

### 3.2. Oral Antibiotics Treatment Enhances IgA Responses to A Multivalent Injected Vaccine

**Figure 2:**
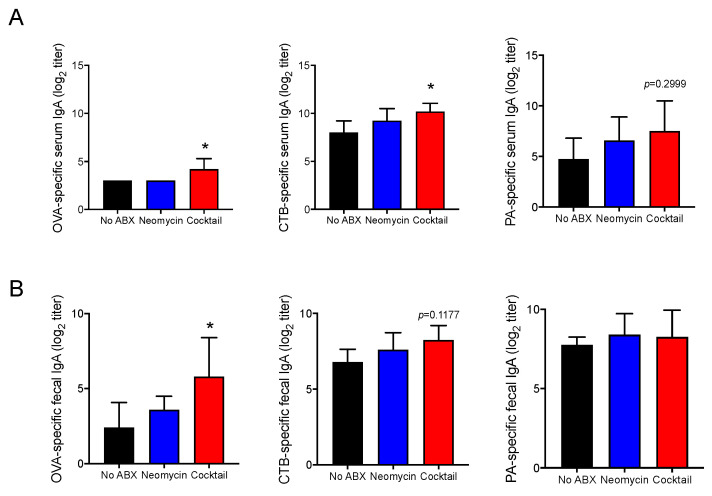
Oral antibiotic treatment enhances IgA responses to a multivalent injected vaccine. Mice were orally treated with antibiotics and then immunized weekly intervals on days 0, 7, and 14 by intraperitoneal injection of the multivalent vaccines. Additional doses of antibiotic were orally administered 24 h before and 24 h after vaccination. (**A**) Serum and (**B**) fecal pellet samples were collected a week after the last immunization (day 21) and antigen-specific IgA titers measured by ELISA. Data are expressed as mean Ab titers ± SD (*n* = 5 mice per group) and are from three independent experiments. * *p* ≤ 0.05 compared to controls non-treated with antibiotic (No ABX).

It was important to determine if antibiotic treatment regulated other immunoglobulin isotype responses. CTB can act as both a vaccine antigen and an adjuvant. But unlike the holotoxin cholera toxin, CTB does not promote IgE responses. Accordingly, neither total nor antigen-specific IgE were induced in mice recipient of our model multivalent vaccine and antibiotics treatment did not promote IgE in these mice (data not shown). Interestingly, the broad-spectrum cocktail of antibiotics enhanced serum IgA responses to CTB and OVA, but not to IgA responses against PA (Figure 2A). A trend toward an increase of IgA responses was also seen with the most antigenic antigens (CTB and PA) in mice treated with the Gram-negative targeting antibiotics (Figure 2A).

We also asked whether the fact that the broad-spectrum cocktail of antibiotics enhanced serum IgA responses could be associated with induction of IgA responses in mucosal tissues. Indeed, we found that OVA-specific IgA responses were increased in the fecal extract of mice treated with the broad-spectrum cocktail of antibiotics (Figure 2B). But as with serum IgG1, different antigens induced different levels of fecal IgA responses (Figure 2B).

### 3.3. Oral Antibiotics Treatment Enhances The Number of IgA Antibody Secreting Cells in The Intestinal Lamina Propria

**Figure 3:**
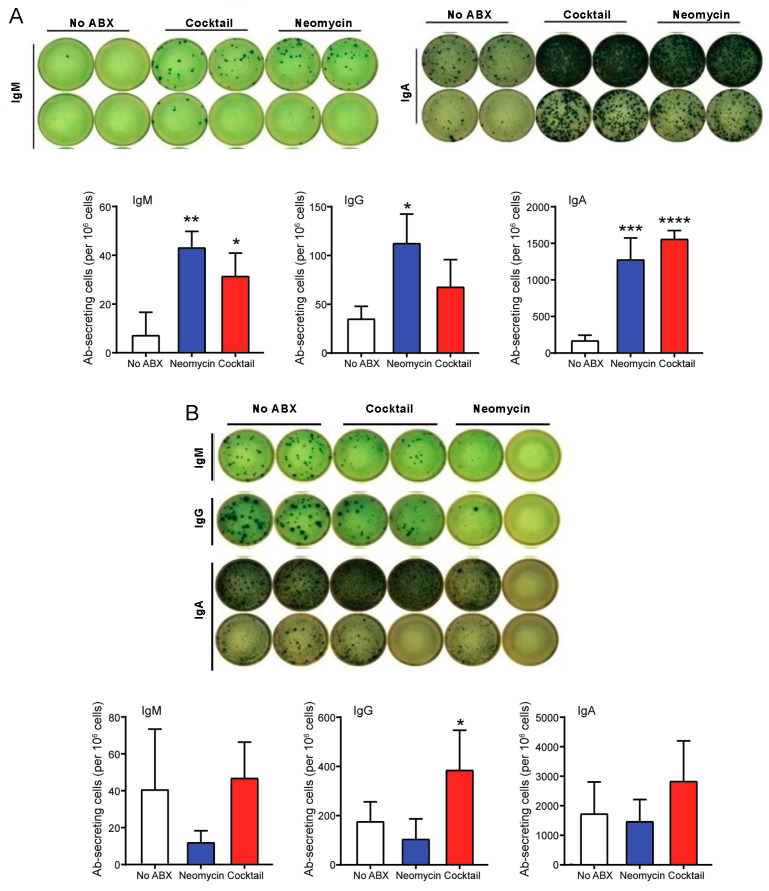
Oral antibiotics treatment enhances the number of IgA antibody secreting cells in the lymph tissues of the gut. Mice were orally treated with antibiotics and then immunized at weekly intervals on days 0, 7, and 14 by intraperitoneal injection of the multivalent vaccines. Additional doses of antibiotics were orally administered 24 h before and 24 h after vaccination. Animals were euthanized 2 weeks after the last immunization (day 28) and immunoglobulin isotype antibody secreting cells (ASC) in the intestinal lamina propria (**A**) and Peyer’s patches (**B**) were measured by ELISPOT. Data are expressed as mean ASC numbers ± SD (*n* = 5 mice per group). * *p* ≤ 0.05; ** *p* ≤ 0.01; *** *p* ≤ 0.001; **** *p* ≤ 0.0001 compared to controls non-treated with antibiotic (No ABX).

Because the magnitude of antigen-specific fecal IgA responses varied between the antigens, we thought to examine whether antibiotic treatment affected the overall number of antibody secreting cells in the intestinal lamina propria. As shown in Figure 3A, both antibiotics treatments significantly increased the frequency of IgM− and IgA−secreting cells. We also noted that the Gram-negative targeting antibiotics significantly increased the frequency of IgG−secreting cells in the small intestinal lamina propria while treatment with the broad-spectrum antibiotics cocktail only induced a trend toward an increase of IgG ASC (Figure 3A).

While intestinal lamina propria are effector sites for production of antibodies that potentially protect gut tissues, antibody-secreting cells can also be found in the Peyer’s patches which are organized lymphoid structures of the gut-associated lymphoid tissues. We found that antibiotics treatment also increased the frequency of IgG− and IgA−ASC in the Peyer’s patches (Figure 3B). Interestingly, this effect was only seen with the cocktail of broad-spectrum antibiotics and not with the antibiotics that targets Gram-negative bacteria (Figure 3B).

### 3.4. Oral Antibiotics Treatment Does Not Increase The Expressing of Gut-homing Receptors in Peripheral Tissues

**Figure 4:**
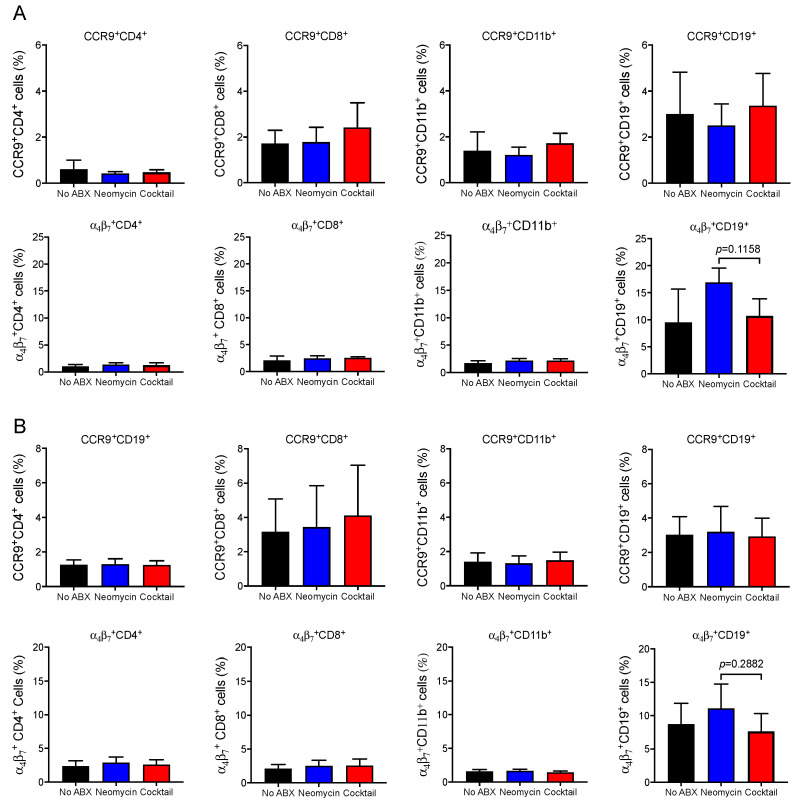
Oral antibiotics treatment does not increase the expressing of gut-homing receptors in peripheral tissues. Mice were orally treated with antibiotics and then immunized weekly intervals on days 0, 7, and 14 by intraperitoneal injection of the multivalent vaccines. Additional doses of antibiotics were orally administered 24 h before and 24 h after vaccination. Animals were euthanized 2 weeks after the last immunization (day 28) and the frequency of spleen cells (**A**) or mesenteric cells (**B**) expressing CCR9 and α_4_β_7_ were determined by flow cytometry after staining with the fluorescent antibodies. Data are expressed as mean ± SD (*n* = 5 mice per group). * *p* ≤ 0.05 compared to controls non-treated with antibiotics (No ABX).

One possible explanation for the increased frequency of IgA ASC in mucosal tissues of mice could be that antibiotics treatment, and more specifically the broad-spectrum antibiotics cocktail, somehow increased the expression of gut-homing receptors. However, analysis of CCR9 and α_4_β_7_ expression by spleen (Figure 4A) and mesenteric (Figure 4B) CD4^+^ and CD8^+^ T cells, B cells (CD19^+^), and myeloid cells (CD11b^+^) showed that neither antibiotics treatment had a significant effect on the expression of these molecules.

### 3.5. Profile of The Gut Microbiome Associated with Increased IgA Responses in Mice Immunized during Oral Antibiotics Treatments

**Figure 5:**
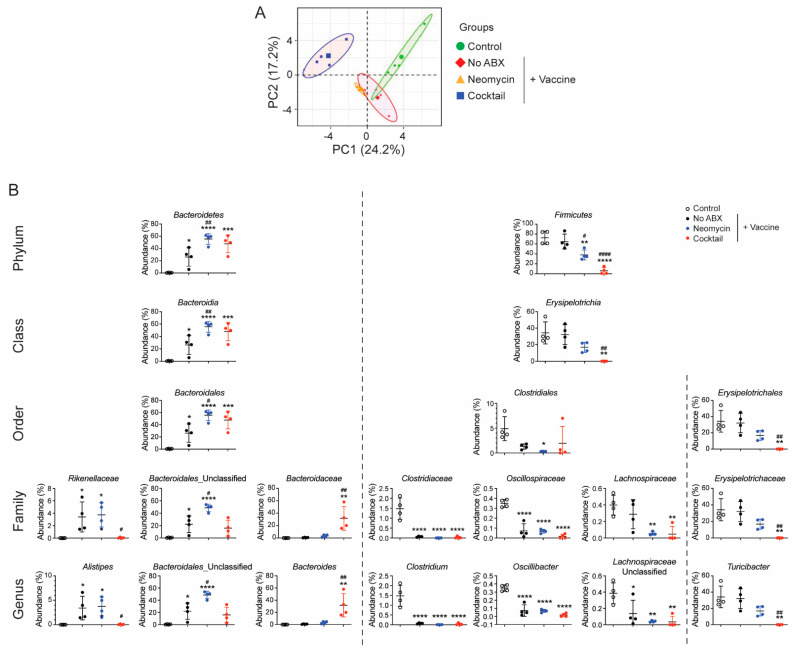
Characterization of gut microbiome of mice treated with oral antibiotics before and throughout vaccination. On day 16, the microbiome present in freshly emitted fecal pellets from control mice, orally administrated saline only (Control: No ABX), or orally treated with a Gram-negative-(Neomycin) or a broad-spectrum antibiotic cocktail (Cocktail) was analyzed by 16s RNA. (**A**) Principal component analysis of bacterial genus in each group. (**B**) Relative abundance of *Firmicutes* and *Bacteroidetes* species at the phylum, class, order, family and genus level. Data are expressed as the mean % abundance ± SD (4 mice per group). * *p* < 0.05; ** *p* < 0.01; *** *p* < 0.001; **** *p* < 0.0001 compared to control. ^#^ *p* < 0.05; ^##^ *p* < 0.01; ^####^ *p* < 0.0001 compared to controls non-treated with antibiotics (No ABX).

Previous studies have shown that selected bacteria strains such as Filamentous bacteria promote IgA responses. Thus, we analyzed the gut microbial community in mice that received the oral antibiotics treatments. Principal component analysis of the bacteria in fecal material confirmed that neomycin which targets Gram-negative bacteria and the broad-spectrum antibiotics cocktail induce distinct profiles of gut microbiota (Figure 5A). We also noted that intragastric gavage of saline (no antibiotics) was enough to alter the gut microbiota, presumably as a consequence of the stress due to frequent manipulation of these animals (Figure 5A). Changes in the ratio of *Firmicutes* to *Bacteroidetes* has been previously used as an indicator of dysbiosis. Consistent with principal component analysis depicted in Figure 5A, intragastric gavage of saline alone (no antibiotic) was sufficient to significantly increase the abundance of *Bacteroidetes*. Nonetheless, gavage of saline alone had no significant effect on the abundance of *Firmicutes* at the Phylum, class or order levels (Figure 5B). Oral treatment with either the neomycin (Gram-negative) or the broad-spectrum antibiotics cocktail reduced the abundance of *Firmicutes* (Figure 5B). But only the broad-spectrum antibiotics cocktail significantly reduced the abundance of *Erysipelotrichales,* including the genus *Turibacter*. On the other hand, the abundance of *Bacteroidetes* was significantly increased by either of the oral antibiotics treatment. However, while neomycin increased the abundance of *Bacteroidales*, the broad-spectrum antibiotic cocktail increased the number of *Bacteroidaceae* (Figure 5B).

**Figure 6:**
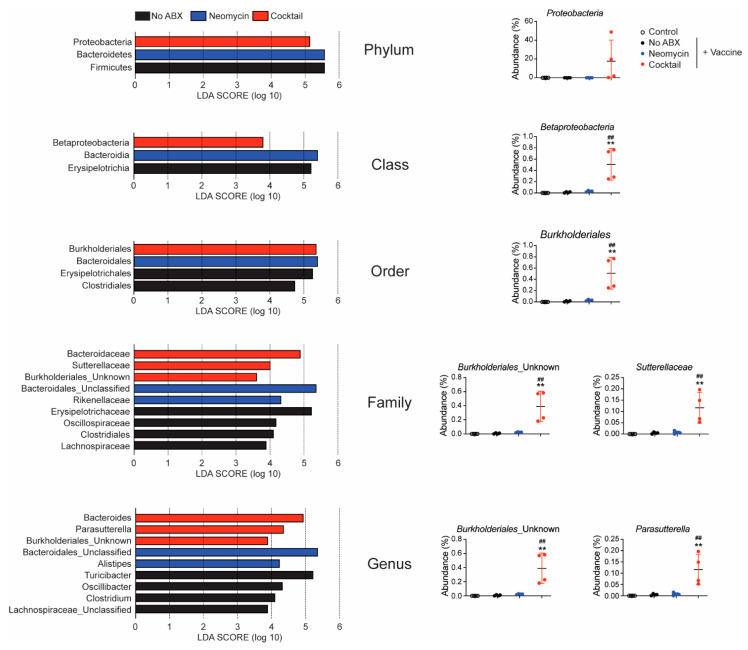
Oral treatment with a large spectrum antibiotics cocktail increases the frequency of *Protebacteria*. On day 16, the microbiome present in freshly emitted fecal pellets from control mice, orally administrated saline only (No Abx), or orally treated with a Gram-negative- (Neomycin) or a broad-spectrum antibiotics cocktail (Cocktail) was analyzed by 16s RNA. Linear discrimination analysis (LDA) scores were analyzed to show the differentially abundant bacteria in each group. Relative abundance of *Proteobacteria* species was found at the phylum, class, order, family, and genus level. Data are expressed as the mean % abundance ± SD (4 mice per group). ** *p* < 0.01 compared to control. ^##^ *p* < 0.01 compared to controls non-treated with antibiotics (No ABX).

Further analysis of the gut microbiome revealed that the broad-spectrum antibiotics cocktail enhanced the abundance of *Proteobacteria*, including *Burkholderiales* and *Sutterellaceae* (Figure 6). This finding is significant since members of the *Proteobacteria* phylum were shown to result in higher serum IgA levels and the induction of large numbers of IgA-secreting plasma cells [35]. Thus, the higher abundance of *Proteobacteria* in mice given the broad-spectrum antibiotics cocktail could explain the higher levels of IgA responses seen in these mice.

## 4. Discussion

Vaccines are among the most significant biomedical achievements since they have reduced the prevalence of several infectious diseases and related mortality. However, although millions of individuals of varying age and health status receive vaccines each year worldwide, antibody and T cell responses to vaccines may vary greatly vary in different individuals. Compelling evidence shows that the efficacy of vaccines can substantially vary with the age, diet, and the microbiota of the recipients. Antibiotics are widely used for the treatment of common infectious agents such as the Gram-negative bacteria *E. coli* and *Salmonella* [36] and it remains unclear how vaccination can be affected by ongoing antibiotics treatments. Here, we report that serum Ig isotype and subclass responses to injected vaccines are differentially regulated by broad-spectrum and Gram-negative-targeting antibiotics. We also show that the gut microbiota induced by oral antibiotics treatments promotes mucosal antibody responses after vaccination via the systemic route.

Commensal microbes stimulate the maturation of the mucosal immune system. Specifically, the development of intestinal Peyer’s patches and the maturation of gut-associated lymphoid tissues are driven by bacterial colonization after birth [37,38]. Commensal microbes also regulate immune responses in the systemic compartment as suggested by reports that germ-free mice or mice lacking the TLR5-sensing of gut microbiota have reduced or impaired serum IgG responses to vaccination [21,22]. Administration of probiotics was shown to improve the immune response to vaccination in germ-free and non-germ-free hosts [39,40,41]. The notion that commensal microbes are an absolute requirement for the development of antibody responses is challenged by the reports that germ-free mice develop higher levels of serum IgE after immunization [6,42]. Taken together, these apparently conflicting reports suggest that the composition of the microbiome or the abundance of selected bacteria differentially regulate the producing of immunoglobulin isotypes. In this regard, segmented filamentous bacteria [43] and members of the *Proteobacteria* phylum [35] were shown to drive production of IgA.

Data summarized in this paper show that IgG1, but not the other IgG subclasses, were regulated by the oral antibiotic treatments. Thus, in order to capture a complete picture of the regulatory effects of the microbiota on systemic IgG responses to vaccines, one should consider the analysis of IgG subclass responses. It is also important to note that only one of the three antigens in our multivalent vaccine had specific IgG1 responses modulated by the microbiome. Our findings are consistent with previous reports that antibiotics treatments have either no effect or only modest effect on IgG responses to vaccines in adults [25,44]. We also provide evidence that the nature of the antigens, and perhaps the difference of immunogenicity between vaccine antigens, could explain the discrepancies between some data reported in the literature. Protection against infections also requires that vaccine antigen-specific antibodies show sufficient affinity for the antigen. The induction of high affinity is achieved with the help of follicular T helper cells [45,46], which can also be affected by the alteration of the microbiome. Thus, future studies could address whether the avidity of antigen-specific IgG subclasses is affected by the antibiotics treatments.

A major finding of this work is the significant effect of antibiotics treatments on IgA responses to all the antigens of our model multivalent vaccine. Thus, both oral antibiotics treatments increased serum IgA responses against all the antigens. However, these IgA responses only reached statistical differences in groups given the broad-spectrum antibiotics cocktail. This finding suggests that IgA responses to injected vaccines are more susceptible to regulation by oral antibiotics treatment than IgG subclasses. The mechanisms underlying these differences remain to be elucidated. Since the production of immunoglobulin classes and subclasses is finely regulated by cytokines [47,48,49], one could speculate that antibiotics treatment increases circulating levels of cytokines that support IgA production in the bloodstream.

The fact that IgA responses were also measured in fecal extracts and that IgA secreting cells were elevated in gut tissues was intriguing because systemic vaccination is generally not effective at promoting immunity in mucosal tissues [12,13]. Homing of effector cells to gut tissues is under the control of mucosal homing receptors α_4_β_7_ and CCR9 [50,51]. The expression of these molecules was surprisingly not significantly increased at the surface of T cells, B cells or myeloid in the spleen or mesenteric lymph node collected after three immunizations. This result could be explained by the time of analysis which was two weeks after the last immunization. Interestingly, the increase in IgA responses seen in mice treated with the broad-spectrum antibiotics is consistent with the increased abundance of *Proteobacteria* which were shown to induce IgA [35]. The abundance of *Bacteroidales* was increased in the fecal pellets of mice treated with either antibiotisc and this could represent a new mechanism that promotes mucosal IgA responses in these mice.

## 5. Conclusions

We have shown that oral treatment of mice with a Gram-negative-targeting antibiotic or broad-spectrum antibiotics cocktail regulates the immune response to vaccine antigens injected in the absence of adjuvant. We have also shown that broad-spectrum antibiotics are more efficient at promoting mucosal IgA responses, in this experimental model. This finding may have important implications in the design of future vaccination strategies against enteric diseases, including those induced by foodborne pathogens.

## Data Availability

The data from the analysis of the gut microbiota will be archived, and shared upon request to the corresponding author.
